# Comparison of various lipid parameters in association of target organ damage: a cohort study

**DOI:** 10.1186/s12944-018-0800-y

**Published:** 2018-08-25

**Authors:** Chen Chi, Jiadela Teliewubai, Yu-Yan Lu, Xi-Min Fan, Shi-Kai Yu, Jing Xiong, Yi-Wu Zhou, Hong-Wei Ji, Yi Zhang, Ya-wei Xu

**Affiliations:** 1 Department of Cardiology, Shanghai Tenth People’s Hospital, Tongji University School of Medicine, 301 Yanchang Road, Shanghai, 200072 China; 2 The Research Institute of Clinical Epidemiology, Tongji University School of Medicine, Shanghai, China

**Keywords:** Target organ damage, Lipid, Lipoprotein, Cholesterol, Primary prevention

## Abstract

**Background:**

Recommendations of non-HDL amplification varied from different guidelines. We aim to test the relationships between various lipid parameters and target organ damage (TOD) including aortic stiffness, peripheral arterial disease and chronic kidney disease in a community-based elderly cohort.

**Methods:**

1599 (aged 71.4 ± 6.1 years) participants were recruited. Eight lipid parameters, including total cholesterol (TC), triglycerides (TG), LDL-C, HDL-C, non-HDL-C, TC/HDL ratio, TG/HDL ratio and LDL/HDL ratio, together with other plasma biomarkers like creatinine were measured. Pulse wave velocity (PWV) was measured by the SphygmoCor device, and ankle-brachial index (ABI) was assessed by Omron VP-1000 device.

**Results:**

Four individual lipid parameters (TC, TG, LDL-C and HDL-C) significantly correlated with most, but not all, TOD indices. Meanwhile, 4 combined lipid parameters, namely non-HDL-C, TC/HDL, TG/HDL and LCL/HDL, significantly correlated with all TOD (*P* ≤ 0.033). In multiple linear regression analyses, 4 combined lipid parameters also significantly associated with TOD (*P* ≤ 0.027), while none of individual lipid parameters significantly associated with all TOD indices. In multiple logistic regression analyses, only non-HDLC and TC/HDL significantly associated with TOD (*P* ≤ 0.039), and other lipid parameters did not significantly associate with TOD.

**Conclusion:**

In an elderly community sample, non-HDLC and TC/HDLC were better associated with TOD than other lipid parameters. This finding should be considered in future clinical lipid-lowing therapy.

**Trial registration:**

This trial was retrospectively registered in ClinicalTrials.gov (No. NCT02368938, registered on 15 Feb 2015).

## Background

Classical lipid profiles, including total cholesterol (TC), triglycerides (TG), low-density lipoprotein (LDL) cholesterol (LDL-C) and high-density lipoprotein (HDL) cholesterol (HDL-C), were identified to play very important roles in cardiovascular diseases (CVD) over past decades. Of these lipid profiles, LDL-C is considered to be one of the strongest predictors of CVD, and lowering LDL-C is fundamental in current CVD prevention and treatment strategies [1–4]. The 2016 European Society of Cardiology guideline for the management of dyslipidemias stated that, LDL-C had to be used in primary lipid analysis and should be the primary target for treatment, while other lipid parameters could be considered in selected cases, for example, those at high risk [4]. However, this LDL-C-based cardiovascular risk assessment and lipid management did not pay much attention to the role of various lipid profiles apart from LDL-C. Numerous studies showed that other lipid profiles, for example HDL, independently associated with CVD [5]. Recent years, there were some attempts to investigate that if these individual lipid parameters could be combined to improve the risk assessment and prediction of cardiovascular events [6]. Non-HDL-C, which calculated as TC - (HDL-C), was found to be another strong independent risk factor of CVD [7]. Based on these findings, the International Atherosclerosis Society guideline recommended that non-HDL-C was as important as LDL-C and non-HDL-C should be the primary targets of therapy since it carried more information of lipids than LDL-C alone [2]. Besides, the recent American National Lipid Association guideline and British National Institute for Health and Care Excellence guideline recommended that non-HDL-C was a better risk indicator than LDL-C [1, 3]. Given the fact that different studies had various population and methodologies, the clinical significance of these combined lipid parameters was still in discussion.

Target organ damage (TOD) is the intermediate stage in the development of CVD, and is a determinant cardiovascular risk factor [8]. Because lipid profiles largely attributed to atherosclerosis, they may closely associate with vascular TOD. Thus, we investigated and compared the individual lipid parameters, which were represented by LDL-C, and the combined lipid parameters, which were represented by non-HDL-C, in association of vascular TOD, aiming to examine the clinical significance of these indices.

## Methods

### Study design and population

The present analysis was based on the “Northern Shanghai Study” (Registry Number: NCT02368938), whose study design and selection criteria has been published before [9]. Briefly, the Northern Shanghai Study is an on-going observational single-center prospective study recruiting community-based elderly subjects (≥65 years old) in the northern Shanghai. Individuals were excluded if they were with serious heart disease (New York Heart Association classification IV), end-stage kidney disease (Chronic kidney disease, CKD ≥4 stage), stroke (within 3 months) or malignant tumor (life expectancy < 5 years). All participants were asked to refrain from food and any vasoactive substance or medication in the morning of the examination. Our present analysis, examining the associations of lipid parameters and TOD, included 1599 participants recruited from the beginning of this study to June, 2015. This study was approved by the local ethics committee and all participants or their legal caregivers voluntarily signed the individual informed consents.

### Medical history and anthropometric measurements

Standardized structured questionnaire was carried out by well-trained physicians to obtain the medical and family history including hypertension, diabetes, drugs, smoking, history of cardiovascular diseases, etc. A trained technician measured body height (in meter) and body weight (in kilogram) of all participants, calculating body mass index (BMI) as body weight divided by the squared body height. After a 10-min rest, another experienced operator assessed blood pressure (BP) 3 times in the sitting position with mercury sphygmomanometer with 3-min interval. The average was calculated and used in subsequent analyses.

### Carotid-femoral pulse wave velocity measurements

According to the European Expert Consensus on Arterial Stiffness [10], the carotid-femoral pulse wave velocity (cf-PWV) was measured by 2 experienced operators with a commonly-used device (SphygmoCor, AtCor Medical, Australia). Before the measurement, each patient was asked to rest for 10 min in a temperature-controlled room, then undergoing BP measurements with an electric BP assessment device (HEM-7211, Omron, Japan). BP were measured twice with 3-min interval in supine position and the average was calculated to be inputted into SphygmoCor device. Distance from the suprasternal notch to common femoral artery (SSN-FA) and the distance from the right carotid artery to suprasternal notch (CA-SSN) were measured. The difference between these two ([SSN-FA]-[CA-SSN]) was regarded as the travelling distance. A sensitive sensor was applied on the right common carotid and the right femoral artery consecutively to detect the pulse waves after the simulation electrocardiogram conduction. If all necessary information, including BP, travelling distance, EKG and pulse waves at two sites, were obtained, the SphygmoCor device automatically calculated cf-PWV and offered an operator index. Only measurements with an operator index over 80% were considered reliable and used in subsequent analysis.

### Ankle-brachial index measurements

One well-trained staff, who was blinded for the study, performed the four-limb blood pressure measurements for each participant once with a validated device (VP-1000, Omron, Japan). The measuring process was performed automatically and simultaneously by this device. Bilateral ankle-brachial index measurements (ABI), defined as the ratio of ankle SBP divided by brachial SBP, was read form this device directly and the lower ABI was actually applied for further analysis.

### Laboratory measurements

Venous blood samples, together with first morning urine samples, were obtained after an overnight fast. TC, TG and HDL-C were measured by standard methods [11, 12], and LDL-C was measured directly to avoid the limitations of Friedewald formula [13]. Given several studies reported that the combined lipid parameters better predicted CV events than individual lipid parameters, the differences of TC and HDL-C (non-HDL), TC/HDL ratio, TG/HDL ratio and LDL/HDL ratio were calculated. Other biological parameters like plasma/urine albumin and creatinine were measured by standard methods at local laboratories. The urine albumin-to-creatinine ratio (UACR) was also calculated.

### Definition of TOD

Asymptomatic TOD related to vascular damage were assessed in present analysis. Aortic stiffness was defined as the cf-PWV over 12 m/s, and participants with ABI lower than 0.9 were considered to be with peripheral arterial disease (PAD). Estimated glomerular filtration rate (eGFR) were calculated based on the Chinese modification of MDRD formula [14], and eGFR< 60 ml/min/1.73 m2 was the cut-off value for the definition of CKD.

### Statistical analysis

Statistical software SAS version 9.3 (SAS Institute, Cary, NC, USA) was used for all statistical analyses. Continuous variables are expressed as mean ± standard deviation (SD) and categorical variables as absolute numbers and percentage. Differences between 2 groups were tested by Student’s t test for continuous variables and by chi-square test for proportions. A two-tailed *P* value < 0.05 was considered statistical significant. Pearson correlation coefficients were calculated to test the bivariate relations of various lipid variables and TOD. Full-model multiple linear and logistic regressions were performed to assess the associations between 1-SD increment in lipid parameters and target organ damage with a full-model method, with one lipid parameter introduced at a time in each model to avoid the collinear effect.

## Results

### Characteristics of participants

Until July 2015, subjects who were over 65-year-old from 10 communities located in the northern Shanghai were screened. One thousand seven hundred twenty-one people were randomly invited, of whom, 1599 subjects participated in this study (responding rate: 92.9%), including 711 (45.5%) men and 843 (52.7%) hypertensive patients. One thousand five hundred eighty-six participants with available data of lipid profiles were finally included in present analysis. Table 1 shows the characteristics of participants and the comparison between men and women.

**Table 1:** General characteristics

	Overall (*n* = 1599)	Men (*n* = 711)	Women (*n* = 888)	*P*
Characteristics				
Ethnics, *n* (%), (Han = 1, Others = 0)	1586 (99.6)	708 (100.0)	878 (99.3)	**0.03**
Body height, cm	159.9 ± 8.3	166.4 ± 6.0	154.7 ± 5.8	**< 0.001**
Body weight, kg	62.4 ± 10.6	67.6 ± 10.2	58.3 ± 8.9	**< 0.001**
Drinker, *n* (%)	237 (14.8)	207 (29.1)	30 (3.4)	**< 0.001**
Cardiovascular risk factors				
Age, years	71.4 ± 6.1	71.3 ± 6.1	71.4 ± 6.1	0.95
Smoker, *n* (%)	366 (22.9)	351 (49.4)	15 (1.7)	**< 0.001**
Body mass index, kg/m^2^	23.9 ± 3.5	23.9 ± 3.3	23.9 ± 3.6	0.92
Fasting plasma glucose, mmol/L	5.69 ± 1.70	5.72 ± 1.67	5.67 ± 1.73	0.55
Systolic blood pressure, mmHg	134.3 ± 17.7	134.3 ± 16.8	134.3 ± 18.4	0.95
Diastolic blood pressure, mmHg	78.9 ± 9.1	80.0 ± 9.2	78.1 ± 9.0	**0.002**
Lipids/lipoproteins				
Total cholesterol, mmol/L	5.22 ± 1.01	4.92 ± 0.99	5.46 ± 0.96	**< 0.001**
Triglycerides, mmol/L	1.61 ± 0.93	1.54 ± 0.85	1.66 ± 1.00	**0.007**
HDL-C, mmol/L	1.38 ± 0.36	1.28 ± 0.33	1.46 ± 0.36	**< 0.001**
LDL-C, mmol/L	3.20 ± 0.85	3.04 ± 0.85	3.33 ± 0.83	**< 0.001**
Non-HDL-C, mmol/L	3.84 ± 0.98	3.65 ± 0.97	4.00 ± 0.96	**< 0.001**
TC/HDL ratio	3.98 ± 1.08	4.05 ± 1.12	3.92 ± 1.06	**0.014**
TG/HDL ratio	1.32 ± 1.05	1.36 ± 0.98	1.30 ± 1.10	0.26
LDL/HDL ratio	2.45 ± 0.81	2.51 ± 0.85	2.39 ± 0.77	**0.005**
Asymptomatic target organ damage				
Pulse wave velocity, m/s	9.42 ± 2.31	9.36 ± 2.42	9.48 ± 2.21	0.32
Creatinine clearance rate, %	92.4 ± 21.7	88.3 ± 20.0	95.8 ± 22.5	**< 0.001**
Urinary albumine-creatinine ratio, mg/g	54.9 ± 181.6	51.1 ± 108.3	58.0 ± 224.4	0.43
Diseases & treatment				
Hypertension, *n* (%)	843 (52.7)	385 (54.2)	458 (51.6)	0.31
Treated hypertension, *n* (%)	799 (93.9)	363 (93.1)	436 (94.6)	0.36
Cardiovascular disease, *n* (%)	549 (34.3)	231 (32.5)	318 (35.8)	0.16
Anti-platelet treatment, *n* (%)	424 (26.6)	204 (28.8)	220 (24.8)	0.07
Lipid lowing therapy, *n* (%)	259 (16.2)	102 (14.4)	157 (17.7)	0.08
Stroke or TIA, *n* (%)	318 (19.9)	130 (18.3)	188 (21.2)	0.15
Renal disease, *n* (%)	132 (8.3)	55 (7.8)	77 (8.7)	0.52
Diabetes, *n* (%)	312 (19.5)	137 (19.3)	175 (19.7)	0.99
Antidiabetic treatment, *n* (%)	262 (16.4)	115 (16.2)	147 (16.6)	0.98
Insulin treatment, *n* (%)	51 (7.4)	22 (7.2)	29 (7.5)	0.90

Data are means ± standard deviation or numbers with percentages in parenthesis. *P* values less than 0.05 were shown in bold type. Creatinine clearance rate was calculated with modified MDRD formula for Chinese. *CVD* Cardiovascular disease, *TIA* Transient ischemia attach. *HDL-C* high-density lipoprotein cholesterol, *LDL-C* low-density lipoprotein cholesterol, *TC* total cholesterol, *TG* triglycerides

The lipid profiles were significantly different between men and women. Men had significantly lower individual lipid parameters, including TC (4.92 ± 0.99 vs 5.46 ± 0.96, *P* < 0.001), TG (1.54 ± 0.85 vs 1.66 ± 1.00, *P* = 0.007), HDL-C (1.28 ± 0.33 vs 1.46 ± 0.36, *P* < 0.001) and LDL-C (3.04 ± 0.85 vs 3.33 ± 0.83, *P* < 0.001). As for combined lipid parameters, compared with women, men had lower non-HDL-C (3.04 ± 0.85 vs 3.33 ± 0.83, *P* < 0.001), higher TC/HDL ratio (4.05 ± 1.12 vs 3.92 ± 1.06, *P* = 0.014), higher LDL/HDL ratio (2.51 ± 0.85 vs 2.39 ± 0.77, *P* = 0.005) and similar TG/HDL ratio (1.36 ± 0.98 vs 1.30 ± 1.10, *P* = 0.26).

### Pearson correlation analyses of lipid parameters and TOD

Pearson correlation analyses were performed to test the correlation between lipid indexes and target organ damage (see Table 2). As for individual lipid parameters, only TG significantly correlated with TOD (*P* ≤ 0.006). None of the other three parameters including TC, HDLC and LDL-C significantly correlated all the TOD. All combined lipid parameters significantly correlated with all the TOD (*P* ≤ 0.033), though the correlation coefficients were not high.

**Table 2:** Correlations of lipid parameters and target organ damage

	PWV		ABI		eGFR	
	R	P	R	P	R	P
TC	0.063	**0.014**	−0.054	**0.032**	−0.006	0.80
TG	0.11	**< 0.001**	− 0.069	**0.006**	−0.086	**< 0.001**
HDL-C	−0.048	0.063	0.11	**< 0.001**	0.13	**< 0.001**
LDL-C	0.051	**0.047**	−0.059	**0.021**	−0.009	0.73
non-HDL-C	0.083	**0.001**	−0.097	**< 0.001**	−0.054	**0.033**
TC/HDL	0.085	**< 0.001**	−0.15	**< 0.001**	−0.13	**< 0.001**
TG/HDL	0.091	**< 0.001**	−0.086	**< 0.001**	−0.11	**< 0.001**
LDL/HDL	0.069	**0.007**	−0.14	**< 0.001**	−0.11	**< 0.001**

Pearson correlation analyses were performed to test the correlation between lipid parameters and target organ damage. *P* values less than 0.05 were shown in bold type. *PWV* pulse wave velocity, *ABI* ankle-brachial index, *eGFR* estimated glomerular filtration rate, *TC* total cholesterol, *TG* triglycerides, *HDL* high density lipoprotein, *LDL* low density lipoprotein

### Linear regression analyses of lipid parameters and TOD

**Fig. 1:**
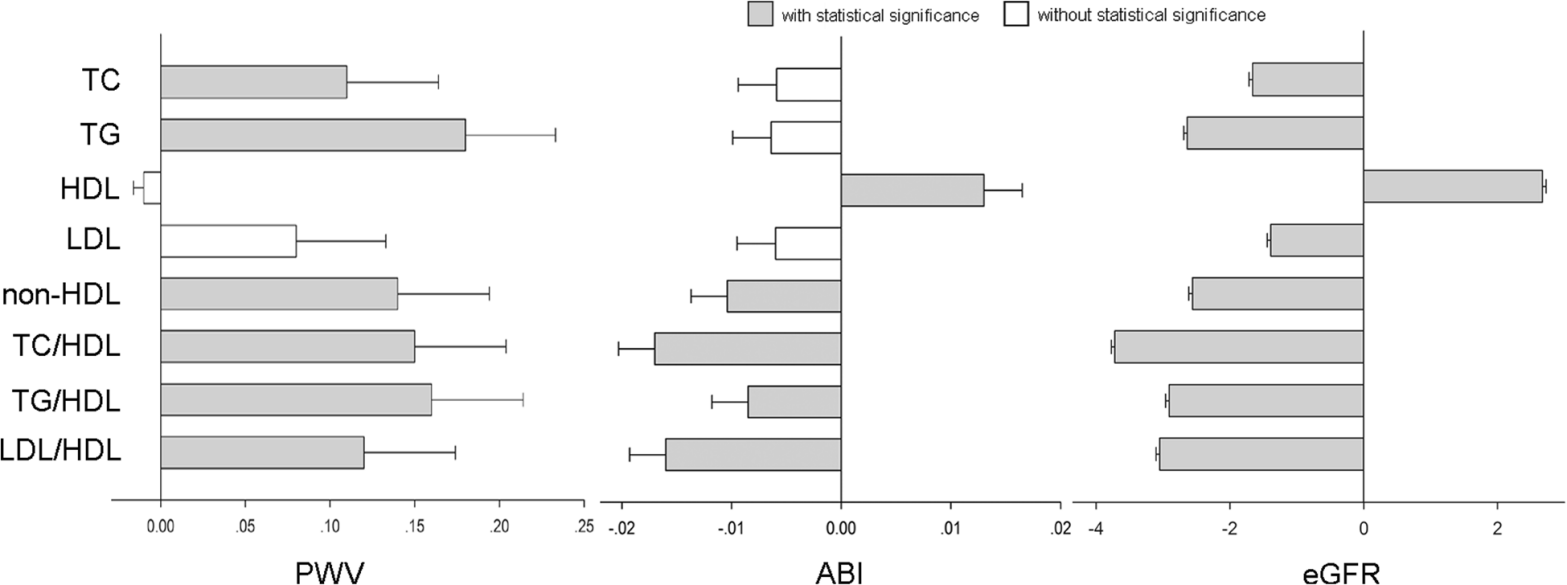
Full-model linear regression analysis of various lipid parameters (as independent variables) and target organ damage: Linear regressions were performed to investigate the association of: (I) pulse wave velocity (PWV), (II) ankle-brachial index (ABI) and (III) estimated glomerular filtration rate (eGFR) with one-SD increment of various lipid parameters respectively. The potential confounders including age, gender, smoke, BMI, fasting blood glucose, systolic blood pressure and lipid lowering therapy, together with lipid parameters, were forced into respective models. Dark color was used to highlight the coefficients achieving statistical significance, and while color represented the coefficients not achieving statistical significance. Only four combined lipid parameters significantly associated with all target organ damage. TC: total cholesterol. TG: triglycerides. HDL: high density lipoprotein. LDL: low density lipoprotein

In Table 3, full-model multiple linear regression analyses were performed to test the association between lipid parameters and TOD. Potential confounders including age, gender, smoking, BMI, fasting blood glucose, systolic blood pressure and lipid lowering therapy were forced into models. After adjustment, none of individual lipid parameters significantly associated with all TOD. TC and TG associated with PWV and eGFR (*P* ≤ 0.04). HDLC associated with ABI and eGFR (*P* < 0.001), and LDL associated with eGFR (*P* = 0.009). However, all combined lipid parameters significantly associated with all the TOD, namely PWV, ABI and eGFR (*P* ≤ 0.01) (Fig. 1).

**Table 3:** Linear regression analyses of lipid parameters and target organ damage

	PWV		ABI		eGFR	
	β ± SE	*P*	β ± SE	*P*	β ± SE	*P*
TC	0.11 ± 0.054	**0.044**	−0.006 ± 0.003	0.060	−1.66 ± 0.54	**0.002**
TG	0.18 ± 0.053	**< 0.001**	−0.006 ± 0.003	0.071	−2.64 ± 0.52	**< 0.001**
HDL-C	−0.01 ± 0.006	0.11	0.013 ± 0.003	**< 0.001**	2.67 ± 0.56	**< 0.001**
LDL-C	0.08 ± 0.053	0.12	−0.006 ± 0.003	0.063	−1.39 ± 0.53	**0.009**
non-HDL-C	0.14 ± 0.054	**0.009**	−0.010 ± 0.003	**0.002**	−2.56 ± 0.53	**< 0.001**
TC/HDL	0.15 ± 0.054	**0.005**	−0.017 ± 0.003	**< 0.001**	−3.72 ± 0.53	**< 0.001**
TG/HDL	0.16 ± 0.054	**0.003**	−0.009 ± 0.003	**0.010**	−2.91 ± 0.53	**< 0.001**
LDL/HDL	0.12 ± 0.054	**0.027**	−0.016 ± 0.003	**< 0.001**	−3.05 ± 0.53	**< 0.001**

Multiple linear regressions were performed to test the associations between each lipid parameter and target organ damage (as continuous variables) after adjustment for age, gender, smoke, BMI, fasting blood glucose, systolic blood pressure and lipid lowering therapy. *P* values less than 0.05 were shown in bold type. *PWV* pulse wave velocity, *ABI* ankle-brachial index, *eGFR* estimated glomerular filtration rate, *TC* total cholesterol, *TG* triglycerides, *HDL* high density lipoprotein, *LDL*: low density lipoprotein

### Logistic regression analyses of lipid parameters and TOD

**Fig. 2:**
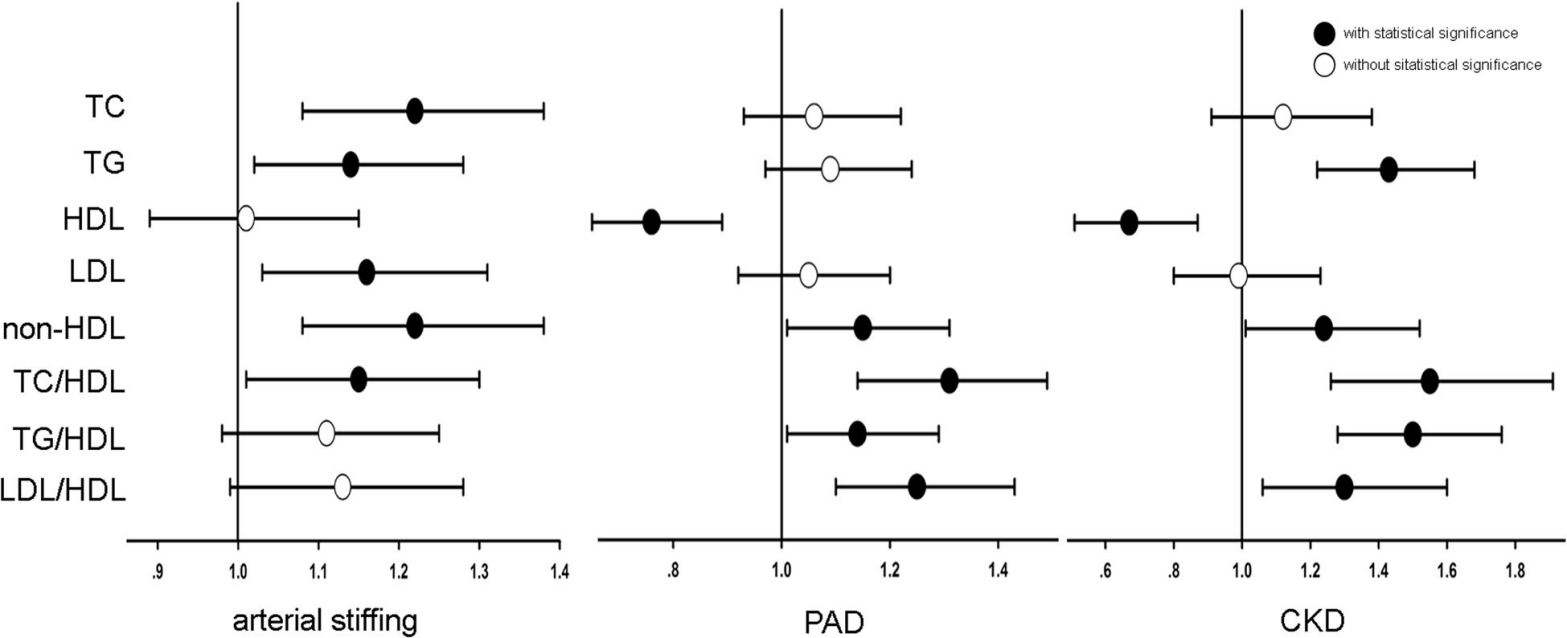
Full-model logistic regression analysis of various lipid parameters (as independent variables) and target organ damage: Logistic regressions were performed to investigate the association of: (I) arterial stiffening, (II) peripheral artery disease (PAD) and (III) chronic kidney disease (CKD) with one-SD increment of various lipid parameters respectively. The potential confounders including age, gender, smoke, BMI, fasting blood glucose, systolic blood pressure and lipid lowering therapy, together with lipid parameters, were forced into respective models. Black circles were used to highlight the odds ratios achieving statistical significance, and while circles represented the odds ratios not achieving statistical significance. Only non-HDL and TC/HDL significantly associated with all target organ damage. Arterial stiffening was defined as pulse wave velocity over 10 m/s. PAD was defined as ankle-brachial index less than 0.9, and CKD was defined as estimated glomerular filtration rate less than 60 mL/min/1.73m^2^. TC: total cholesterol. TG: triglycerides. HDL: high density lipoprotein. LDL: low density lipoprotein

In Table 4, full-model multiple logistic regression analyses were performed to test the association between lipid parameters and TOD. After the same adjustment for potential confounders including age, gender, smoking, BMI, fasting blood glucose, systolic blood pressure and lipid lowering therapy, only non-HDL and TC/HDL significantly associated with all TOD (*P* ≤ 0.04). Neither individual lipid parameters, nor the other 2 combined lipid parameters, significantly associated with all TOD (Fig. 2).

**Table 4:** Logistic regression analyses of lipid parameters with target organ damage

	Arterial Stiffness		PAD		CKD	
	OR [95% CI]	*P*	OR [95% CI]	*P*	OR [95% CI]	*P*
TC	1.22 [1.08, 1.38]	**0.002**	1.06 [0.93, 1.22]	0.37	1.12 [0.91, 1.38]	0.30
TG	1.14 [1.02, 1.28]	**0.026**	1.09 [0.97, 1.24]	0.16	1.43 [1.22, 1.68]	**< 0.001**
HDL-C	1.01 [0.89, 1.15]	0.90	0.76 [0.65, 0.89]	**< 0.001**	0.67 [0.51, 0.87]	**0.003**
LDL-C	1.16 [1.03, 1.31]	**0.016**	1.05 [0.92, 1.20]	0.47	0.99 [0.80, 1.23]	0.96
non-HDL-C	1.22 [1.08, 1.38]	**0.002**	1.15 [1.01, 1.31]	**0.043**	1.24 [1.01, 1.52]	**0.039**
TC/HDL	1.15 [1.01, 1.30]	**0.030**	1.31 [1.14, 1.49]	**< 0.001**	1.55 [1.26, 1.91]	**< 0.001**
TG/HDL	1.11 [0.98, 1.25]	0.091	1.14 [1.01, 1.29]	**0.040**	1.50 [1.28, 1.76]	**< 0.001**
LDL/HDL	1.13 [0.99, 1.28]	0.060	1.25 [1.10, 1.43]	**< 0.001**	1.30 [1.06, 1.60]	**0.013**

Full-model logistic regressions were performed to test the associations between each lipid parameter and target organ damage (as binary variables) after adjustment for age, gender, smoke, BMI, fasting blood glucose, systolic blood pressure and lipid lowering therapy. *P* values less than 0.05 were shown in bold type. *PAD* peripheral arterial disease, *CKD* chronic kidney disease, *TC* total cholesterol, *TG* triglycerides, *HDL* high density lipoprotein, *LDL* low density lipoprotein

## Discussion

The relations of individual and combined lipid parameters with vascular TOD were compared in this study. The results showed that, in a community-based elderly cohort, combined lipid parameters, especially non-HDL and TC/HDL, were better associated with vascular TOD than individual lipid parameters.

LDL-C, as the representative of classical individual lipid parameters, is a widely accepted independent cardiovascular risk factor and is almost included in all cardiovascular risk estimation tools [4]. Though LDL-C is very important in atherogenesis, it only accounts for about 75% atherogenic lipoproteins, while the other 25% like very low density lipoprotein (VLDL) also contribute to this progression [15]. Recent years, emerging data showed that, combined lipid parameters had strong power in CVD prediction. Especially non-HDL-C, which carried the information reflecting not only LDL, but also VLDL, intermediate-density lipoprotein, lipoprotein (a), etc., seemed to be superior than LDL-C [16]. Several studies reported that non-HDL-C could better predict cardiovascular events than LDL-C [6, 17, 18], and meta-analysis also showed that the non-HDL-C was better associated with future cardiovascular events among statin-treated patients [7]. The superiority of non-HDL-C may be owning to several reasons. For example, not only LDL but also other lipoproteins enter the arterial wall and contribute to atherogenesis [16]. Besides, non-HDL-C better correlates with apolipoprotein B (apo B), which is a direct indicator of nearly all atherogenic lipoproteins [19].

Though non-HDL-C seemed to be superior over LDL-C particularly in those with high TG, some guidelines did not recommend it as primary target in dyslipidemia management. The major concern was that though non-HDL-C performed very well in epidemiologic studies, the support of the use of non-HDL-C instead of LDL-C was weak in random controlled trials (RCTs) [4]. And till now, there is no large RCT comparing the treatment strategies based on LDL-C or non-HDL-C respectively [2]. In meta-analysis, the absolute difference between the associations of non-HDL-C and LDL-C with cardiovascular events was significant but slight [7]. From our point of view, it is partly owning to the difference of populations. Most RCTs focused on treatment, which meant the participants were dyslipidemia patients, in another word, they were in “abnormal” conditions. However, epidemiologic studies containing both “normal” and “abnormal” participants, reflecting not only pathological but also physiological conditions. According to the famous cardiovascular event chain proposed by Dzau and Braunwald, the development of CVD is a continuum [20], and TOD is an important intermediate stage in this progression. In our study, TOD was independently associated with non-HDL-C. This result together with the results that we mentioned above indicated that, non-HDL-C might be a better primary target than LDL-C among people at risk of developing CVD. For patients with CVD, both non-HDL-C and LDL-C should be taken into consideration since all trials used LDL-C and the role of LDL-C was well established.

In our study, TC/HDL was another lipid parameter which associated with vascular TOD. As early as 1992, Castelli et al. reported that, apart from LDL-C, TC/HDL ratio was another powerful independent predictor of cardiovascular events in Framingham Study [21]. Several studies tried to compare the effects of non-HDL-C and TC/HDL, but the difference was too little to distinguish these two lipid parameters [22, 23]. Given the fact that non-HDL-C and TC/HDL showed different aspects of the relationship of atherogenic and anti-atherogenic lipid profiles, namely the absolute difference and the ratio, the role of TC/HDL might be of great importance in risk stratification and treatment in the future.

There were some limitations in this study. As a cross-sectional study, we could not provide more information other than associations. However, the Northern Shanghai Study is an on-going study and we are doing the follow-up. Hopefully, we could have more interesting findings in the future. Second, we did not measure other lipid parameters such as apo B, which also showed its superiority over LDL-C in some studies [7]. Third, the present analysis did not include the analysis on intima-media thickness and carotid plaque, which had been regarded as an important marker of vascular TOD for several decades. However, emerging data showed that intima-media thickness might not be a good marker of TOD. Fourth, it should be pointed out that though the associations between TOD and lipid parameters were significant in our study, the correlation coefficients were not high, suggesting that TOD was a multi-factor-driven process.

## Conclusion

The present analysis showed that, in this community-dwelling elderly cohort, non-HDLC and TC/HDLC were better associated with vascular TOD including arterial stiffening, PAD and CKD. This finding should be considered in future clinical practice and dyslipidemia management. Large clinical trials, especially RCTs directly comparing LDL-C and non-HDL-C as treatment target, are warranted in the future.

## Abbreviations

ABI: Ankle-brachial index
BMI: Body mass index
BP: Blood pressure
CVD: Cardiovascular diseases
eGFR: Estimated glomerular filtration rate
HDL: High-density lipoprotein
LDL: Low-density lipoprotein
PAD: Peripheral arterial disease
PWV: Pulse wave velocity
RCT: Random controlled trials
SD: Standard deviation
TC: Total cholesterol
TG: Triglycerides
TOD: Target organ damage
UACR: Urine albumin-to-creatinine ratio
VLDL: Very low density lipoprotein
